# HIV Coinfection Is Associated with Low-Fitness *rpoB* Variants in Rifampicin-Resistant Mycobacterium tuberculosis

**DOI:** 10.1128/AAC.00782-20

**Published:** 2020-09-21

**Authors:** Chloé Loiseau, Daniela Brites, Miriam Reinhard, Kathrin Zürcher, Sonia Borrell, Marie Ballif, Lukas Fenner, Helen Cox, Liliana K. Rutaihwa, Robert J. Wilkinson, Marcel Yotebieng, E. Jane Carter, Alash'le Abimiku, Olivier Marcy, Eduardo Gotuzzo, Anchalee Avihingsanon, Nicola Zetola, Basra Doulla, Erik C. Böttger, Matthias Egger, Sebastien Gagneux

**Affiliations:** a Swiss Tropical and Public Health Institute, Basel, Switzerland; b University of Basel, Basel, Switzerland; c Institute of Social and Preventive Medicine, University of Bern, Bern, Switzerland; d Institute of Infectious Disease and Molecular Medicine and Wellcome Centre for Infectious Disease Research in Africa, University of Cape Town, Cape Town, South Africa; e Ifakara Health Institute, Bagamoyo, Tanzania; f Wellcome Centre for Infectious Diseases Research in Africa, University of Cape Town, Observatory, South Africa; g Department of Infectious Diseases, Imperial College London, London, United Kingdom; h Francis Crick Institute, London, United Kingdom; i Division of General Internal Medicine, Department of Medicine, Albert Einstein College of Medicine, Bronx, New York, USA; j Department of Medicine, Moi University School of Medicine, and Moi Teaching and Referral Hospital, Eldoret, Kenya; k Institute of Human Virology, Abuja, Nigeria; l Centre de Prise en Charge de Recherche et de Formation, Yopougon, Abidjan, Côte d'Ivoire; m Bordeaux Population Health Research Center, Inserm U1219, University of Bordeaux, Bordeaux, France; n TB Research Unit, Instituto de Medicina Tropical Alexander von Humboldt, Universidad Peruana Cayetano Heredia, Lima, Peru; o The HIV Netherlands Australia Thailand (HIV-NAT) Research Collaboration, Thai Red Cross AIDS Research Centre and Tuberculosis Research Unit, Faculty of Medicine, Chulalongkorn University, Bangkok, Thailand; p University of Pennsylvania, Philadelphia, Pennsylvania, USA; q Central Tuberculosis Reference Laboratory, Dar es Salaam, Tanzania; r National Tuberculosis and Leprosy Programme, Dar es Salaam, Tanzania; s Institute of Medical Microbiology, University of Zurich, Zurich, Switzerland; t Swiss National Center for Mycobacteria, Zurich, Switzerland; u Centre for Infectious Disease Epidemiology and Research, Faculty of Health Sciences, University of Cape Town, Cape Town, South Africa

**Keywords:** HIV-TB coinfection, *Mycobacterium tuberculosis*, drug resistance, fitness cost, rifampicin

## Abstract

We analyzed 312 drug-resistant genomes of Mycobacterium tuberculosis isolates collected from HIV-coinfected and HIV-negative TB patients from nine countries with a high tuberculosis burden. We found that rifampicin-resistant M. tuberculosis strains isolated from HIV-coinfected patients carried disproportionally more resistance-conferring mutations in *rpoB* that are associated with a low fitness in the absence of the drug, suggesting these low-fitness *rpoB* variants can thrive in the context of reduced host immunity.

## INTRODUCTION

Tuberculosis (TB), caused by members of the Mycobacterium tuberculosis complex, is a leading cause of death worldwide, killing more people than any other infectious disease. Among the many factors driving the global TB epidemics, two factors stand out as particularly important: antibiotic resistance and HIV coinfection (1). Although the impact of both of these factors individually is well recognized, the interaction between them is less clear and likely depends on the particular epidemiologic setting (2). HIV coinfection and drug-resistant TB often coexist in severe epidemics, which indicates spread of drug-resistant M. tuberculosis strains from immunocompromised patients (3–5). The propensity of drug-resistant M. tuberculosis strains to spread is influenced by the fitness cost associated with drug resistance determinants (6). Specifically, bacterial strains that have acquired drug resistance-conferring mutations may be less transmissible than their susceptible counterparts, although this fitness cost can be ameliorated by compensatory mutations (7–10). Moreover, the effect of different resistance-conferring mutations on fitness can be heterogeneous (11). In the clinical setting, there is a selection for high-fitness and/or compensated drug-resistant M. tuberculosis strains in TB patients (12). However, in immunocompromised hosts, such as HIV-coinfected patients, even strains with low-fitness resistance mutations might propagate efficiently (13–15), which could partially explain why drug-resistant TB has been associated with HIV coinfection (16, 17). However, to date, no evidence directly supports the notion that the immunological environment created by HIV coinfection modifies the fitness of drug-resistant M. tuberculosis (5, 18, 19).

**FIG 1:**
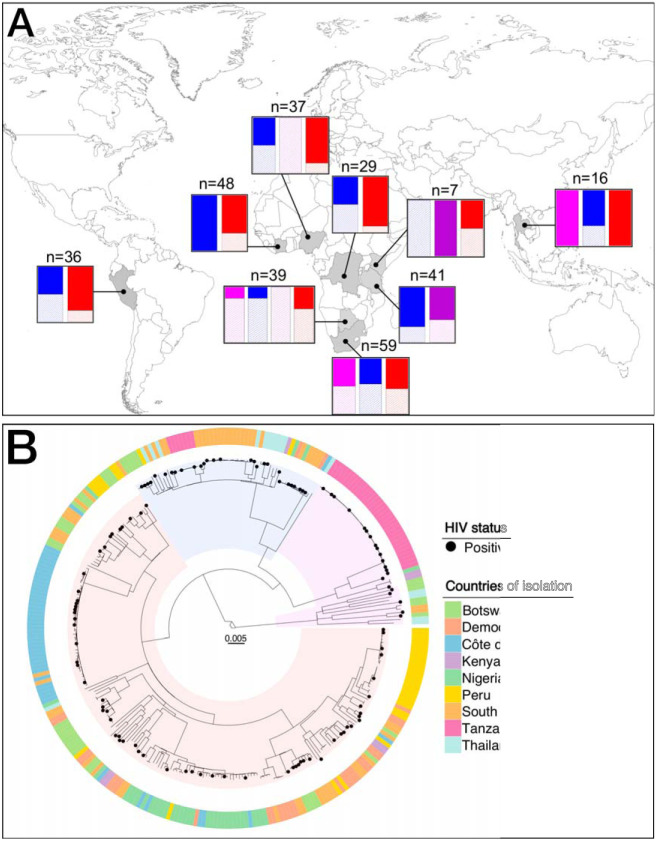
(A) Frequency of M. tuberculosis lineages by HIV status for countries sampled. Countries colored in gray were sampled. The bar plots indicate the proportion of each lineage represented in this study. Magenta corresponds to M. tuberculosis lineage 1, blue corresponds to M. tuberculosis lineage 2, purple corresponds to M. tuberculosis lineage 3, and red corresponds to M. tuberculosis lineage 4. Solid color corresponds to HIV-negative patients, and hatches correspond to HIV-coinfected TB patients. The number of genomes sampled in each country is indicated on top of the bar plots. (B) Phylogenetic tree of the data set used in the study. Maximum likelihood phylogeny of 312 whole-genome sequences based on 18,531 variable positions. The scale bar indicates the number of substitutions per polymorphic site. The phylogeny was rooted on *Mycobacterium canettii*. M. tuberculosis strains isolated from HIV-coinfected patients are indicated by black dots. The peripheral ring depicts the country of isolation of the strains sequenced.

In this study, we tested the hypothesis that resistance-conferring mutations with low fitness in M. tuberculosis are overrepresented among HIV-coinfected TB patients. We focused our analysis on isoniazid and rifampicin, the two most important first-line anti-TB drugs, for which resistance-conferring mutations have been shown to differ in their fitness effects when measured in the laboratory (11). In addition, the frequency of the resistance alleles found in a clinical setting correlates well with the *in vitro* fitness of strains (12, 20). To explore the association between HIV coinfection and the fitness effect of different drug resistance-conferring mutations in M. tuberculosis, we compiled a collection of drug-resistant strains using the global International Epidemiology Databases to Evaluate AIDS (IeDEA, http://www.iedea.org) consortium (21, 22) as a platform. For this study, 312 strains were collected from HIV-coinfected and HIV uninfected TB patients originating from nine countries on three continents: Peru, Thailand, South Africa, Kenya, Côte d’Ivoire, Botswana, Democratic Republic of the Congo, Nigeria, and Tanzania (Fig. 1; see also Table S1 in the supplemental material). The association between the fitness of isoniazid resistance-conferring mutations and HIV coinfection was tested in a univariate analysis (Fig. S1). Isoniazid resistance-conferring mutations were divided into three groups, as previously described (23): *katG* S315T mutation, *katG* mutations other than S315T, and *inhA* promoter mutations only. The S315T substitution in *katG* causes high-level isoniazid resistance while retaining some catalase/peroxidase functions (24). Conversely, the *inhA* promoter mutation does not affect KatG activity. Other substitutions/deletions in *katG* have been associated with a lower fitness in the laboratory and are observed only rarely among clinical isolates (23, 25, 26). In the case of rifampicin, the association between the fitness of *rpoB* variants and HIV coinfection was tested in both a univariate and multivariate analysis (Table 1). Resistance-conferring variants in *rpoB* were classified into two groups based on their fitness effects documented previously (11, 20, 27). The mutation *rpoB* S450L was considered high fitness, since this mutation was previously shown to confer a low fitness cost in the laboratory (11) and is generally the most common in clinical strains (28). Any other resistance-conferring variant affecting *rpoB* was considered low fitness (11). The multivariable logistic regression model with outcome of low-fitness *rpoB* variants was adjusted for host-related factors (history of TB, country of isolation, sex, and age) (29) and bacterial factors (M. tuberculosis lineage, presence of an *rpoA-C* compensatory mutation, clustering of the genome inferred by genetic relatedness). Seventy-six patients from Tanzania and Botswana were excluded from the model due to missing or unknown clinical data (see the supplemental methods file).

**TABLE 1:** Results of the univariate and multivariate analysis showing host and bacterial factors associated with low fitness *rpoB* variants in 203 TB patients^*a*^

Parameter for fitness of *rpoB* variants	No. (%) of patients by fitness level		Univariable		Multivariable	
	Low	High	OR (95% CI)	*P* value	OR (95% CI)	*P* value
HIV status						
HIV^−^	71 (51.4)	67 (48.6)	Reference		Reference	
HIV^+^	47 (72.3)	18 (27.7)	2.46 (1.30–4.66)	0.006	4.58 (1.69–12.44)	0.003
Presence of a compensatory mutation in *rpoA-C*						
No	117 (71.3)	47 (28.7)	Reference		Reference	
Yes	1 (2.6)	38 (97.4)	0.01 (0.00–0.08)	< 0.0001	0.01 (0.00–0.06)	< 0.0001
M. tuberculosis lineage						
2	16 (44.4)	20 (55.6)	Reference		Reference	
4	99 (61.5)	62 (38.5)	2.00 (0.96–4.14)	0.06	3.10 (0.94–10.21)	0.06
Other (L1 or L3)	3 (50.0)	3 (50.0)	1.25 (0.22–7.05)	0.80	0.97 (0.11–8.31)	0.98
Clustering of the genome						
No	109 (59.6)	74 (40.4)	Reference		Reference	
Yes	9 (45.0)	11 (55.0)	0.56 (0.22–1.41)	0.21	1.05 (0.28–3.90)	0.94
Country of isolation						
South Africa	29 (55.8)	23 (44.2)	Reference		Reference	
Democratic Republic of Congo	11 (37.9)	18 (62.1)	0.48 (0.19–1.23)	0.13	0.39 (0.12–1.34)	0.14
Côte d'Ivoire	35 (79.5)	9 (20.5)	3.08 (1.24–7.70)	0.02	2.04 (0.58–7.23)	0.27
Kenya	4 (66.7)	2 (33.3)	1.59 (0.27–9.44)	0.61	0.94 (0.10–8.42)	0.96
Nigeria	20 (58.8)	14 (41.2)	1.13 (0.47–2.72)	0.78	1.00 (0.29–3.40)	0.99
Peru	16 (53.3)	14 (46.7)	0.91 (0.37–2.23)	0.83	1.49 (0.33–6.70)	0.60
Thailand	3 (37.5)	5 (62.5)	0.48 (0.10–2.20)	0.34	0.42 (0.07–2.65)	0.36
Age						
Mean (SD)	32.5 (10.4)	34.3 (12.3)	0.99 (0.96–1.01)	0.25	0.97 (0.94–1.01)	0.10
Sex						
Female	47 (59.5)	32 (40.5)	Reference			
Male	71 (57.3)	53 (42.7)	0.91 (0.51–1.62)	0.75	0.77 (0.34–1.71)	0.52
History of TB disease						
No	35 (52.2)	32 (47.8)	Reference			
Yes	83 (61.0)	53 (39.0)	1.43 (0.79–2.58)	0.23	0.96 (0.34–2.73)	0.94

a Number of observations in model, 203; CI, confidence interval. The odds ratios and *P* values were obtained from the regression model.

Out of 312 patients, 113 (36.2%) were HIV coinfected, 120 (38.5%) were women, 115 (36.9%) were newly diagnosed TB cases (therefore, treatment naive), 276 (88.5%) harbored isoniazid resistance-conferring mutations, with or without additional resistance, and 282 (90.4%) harbored rifampicin resistance-conferring mutations, with or without additional resistance. In total, 78.8% (*n* = 246) of the strains were classified as being at least multidrug resistant, defined as resistance to isoniazid and rifampicin with or without additional resistance to second-line drugs. Among the 113 HIV-coinfected individuals, 34 (30%) were on antiretroviral therapy (ART), 26 (23%) were not, and 53 (47%) had an unknown ART start date. Four of the eight known M. tuberculosis lineages were represented in the following proportions: 11 L1 (3.5%), 57 L2 (18.3%), 38 L3 (12.2%), and 206 L4 (66.0%). After dividing a total of 276 isoniazid-resistant strains into the three groups of isoniazid resistance-conferring mutations defined above, we found similar proportions in HIV-coinfected and HIV-uninfected patients (chi-square test, *P* = 0.54; Fig. S1), and, as expected, the *katG* S315T mutation was the most frequent mutation in both categories (overall, found in 80% of isoniazid-resistant strains). In the case of rifampicin resistance, a univariate and multivariate analysis of 203 strains with complete clinical records indicated that HIV-coinfected TB patients carried a higher proportion of low-fitness *rpoB* resistance variants than HIV-negative patients (72.3% versus 51.4%). The univariate analysis showed higher odds of having a low-fitness *rpoB* variant in HIV-coinfected patients (odds ratio, 2.46 [95% confidence interval, 1.30 to 4.66], *P* = 0.006) (Table 1). Our multivariable regression analysis confirmed these results and showed an association between low-fitness *rpoB* variants and HIV coinfection while controlling for other factors (odds ratio, 4.58 [95% confidence interval, 1.69, 12.44], *P* = 0.003) (Table 1). This association can be explained in at least two ways. First, HIV-coinfected patients are thought to have fewer lung cavities on average and lower sputum bacillary load (30, 31). The resulting smaller M. tuberculosis population size would lead to fewer replication events, possibly reducing the number of mutations available for selection to act upon. In other words, low-fitness variants and high-fitness variants would co-occur less often in an HIV-coinfected patient, such that competition between them would be less likely. This scenario would be relevant for *de novo* acquisition of low-fitness drug-resistant variants within an HIV-coinfected patient. Second, following the transmission of a drug-resistant strain with low fitness to a host with reduced immunity, weaker immune pressure acting on this strain might lead to better bacterial survival. The association between low-fitness *rpoB* variants and HIV coinfection remained significant even after adjusting for the different epidemiologic settings (i.e., countries) and the strain genetic background (i.e., M. tuberculosis lineages). We also observed that strains carrying the *rpoB* S450L resistance-conferring mutation were more likely to also carry a compensatory mutation in *rpoA-C* (97.4% versus 2.6%) (Table 1). Even though this phenomenon seems counterintuitive, it has been described multiple times (7, 9, 32–34) and, thus, might point to different mechanisms of compensation in strains carrying resistance mutations other than *rpoB* S450L. In addition, in our study, L4 strains were associated with low-fitness *rpoB* variants compared to L2 (odds ratio, 3.10 [95% confidence interval, 0.94, 10.21], *P* = 0.06) (Table 1), indicating that the strain genetic background plays a role in shaping the cost of resistance, as was previously shown for other bacterial species (35) and for other drugs (36). In the regression analysis, we had several categorical variables with only a few observations. Therefore, statistical power, especially for country of isolation, was low, and the results should be interpreted with care.

HIV-coinfected TB patients are generally thought to have a reduced potential for TB transmission (30, 37), because these patients have reduced formation of lung cavities, more extrapulmonary disease, and a shorter period of infectiousness due to earlier diagnosis or higher mortality, especially in the absence of antiretroviral treatment and if antibiotic resistance is already present (4). Based on the overrepresentation of low-fitness *rpoB* mutations in the context of HIV coinfection, one would expect a further reduction of the transmission potential of drug-resistant TB in this context. However, outbreaks of drug-resistant TB in HIV-coinfected patients have been reported (3). Such outbreaks might be explained by (i) a higher risk of M. tuberculosis infection and reinfection due to diminished host immunity, (ii) on-going transmission of drug-resistant M. tuberculosis from a larger pool of immunocompetent TB patients to immunocompromised patients, (iii) transmission occurring in conducive environments, such as health care settings, where both HIV-coinfected individuals and drug-resistant TB patients are more likely to coexist, and (iv) M. tuberculosis strains carrying high-fitness drug resistance mutations.

In summary, using a global sample of drug-resistant M. tuberculosis clinical strains from HIV-coinfected and HIV-negative TB patients, we showed that low-fitness *rpoB* variants were overrepresented in HIV-coinfected patients, and that this association was independent from other potential confounding factors. Taken together, our results provide new insights into how HIV coinfection can impact the fitness of drug-resistant M. tuberculosis.

## 

### Data availability.

The M. tuberculosis whole-genome sequences from the patients are available on NCBI under several project identifiers. The accession number for each genome is indicated in Supplemental Table S1.

## Supplementary Material

Supplemental file 1

Supplemental file 2
