# Supplementary material for: HIV Coinfection Is Associated with Low-Fitness rpoB Variants in Rifampicin-Resistant Mycobacterium tuberculosis
Source: Antimicrob Agents Chemother. 2020 Sep 21;64(10):e00782-20. doi: 10.1128/AAC.00782-20 (PMC7508592; doi:10.1128/AAC.00782-20)
Supplement: Supplemental file 1 [file AAC.00782-20-s0001.pdf]

Supplement for "HIV coinfection is associated with low fitness *rpoB* variants in rifampicin-resistant *Mycobacterium tuberculosis*"

Methods

Data collection: Participants were recruited prospectively from January 2013 to December 2016, as described in Zürcher et al (1). Patients aged 14 years or above and who were treated for active pulmonary tuberculosis in Côte d'Ivoire, Democratic Republic of the Congo, Botswana, Tanzania, Kenya, Nigeria, South Africa, Peru, and Thailand were included. HIV-positive patients with tuberculosis were recruited prospectively from ART clinics participating in IeDEA and HIV-negative patients were recruited prospectively from tuberculosis clinics serving the same population. In South Africa, recruited patients came from strain collections held at the University of Cape Town. Patients received TB treatment according to local drug-susceptibility testing. However the isolates were collected before TB treatment started.

Whole genome Sequencing: Strains were cultured on Löwenstein-Jensen slants and purified DNA for WGS was obtained using the CTAB extraction method (2). Libraries were prepared using the Illumina Nextera XT kit and sequenced on an Illumina HiSeq 2500. Sequencing was performed at the genomics facility of the ETHZ, Switzerland and at the Broad Institute, Cambridge, Massachusetts, United States.

Genomics analysis: Raw genomic reads were trimmed with Trimmomatic v0.33 (3). Only reads larger than 20bp were kept for the downstream analysis. SeqPrep (https://github.com/jstjohn/SeqPrep) was used to identify and merge any overlapping paired-end reads. The resulting reads were aligned to the reconstructed ancestral sequence of the *M. tuberculosis* complex using the mem algorithm of BWA v0.7.13(4). Duplicated reads were marked using the MarkDuplicates module of Picard v2.9.1 (https://github.com/broadinstitute/picard). The RealignerTargetCreator and IndelRealigner modules of GATK v3.4.0 were used to perform local realignment of reads around InDels (5). Single nucleotide polymorphisms (SNPs) were called with Samtools v1.2 mpileup (6) and VarScan v2.4.1 (7) using a minimum mapping quality of 20, minimum base quality at a position of 20, minimum read depth at a position of 7X, maximum strand bias for a position

90%. SNPs were annotated using snpEff v4.1144 (8), using the *M. tuberculosis* H37Rv reference annotation (NC_000962.3). Resistance-conferring variants were screened in the resulting VCF files and only variants with a frequency of ≥ 80% were considered.

Categorization of rpoB *mutations:* 282/312 strains harbored rifampicin resistance-conferring variants. The different *rpoB* mutations are reported in the supplementary table 1. The mutation *rpoB* S450L was considered 'high-fitness', whereas any other resistance-conferring mutation affecting *rpoB* was considered 'low-fitness'._23/282 strains carried 2 different *rpoB* mutations. In the cases where one of the two *rpoB* mutations was a S450L mutation, the strain was categorized in the group 'high-fitness *rpoB* mutation'. Conversely, when none of the two mutations was a S450L, the strain was categorized in the group 'low-fitness *rpoB* mutation'.

Selection of 203 genomes used in the logistic regression: the association between the fitness of *rpoB* variants and HIV coinfection was tested in both a univariate and multivariate analysis (Table 1). Therefore, only the genomes harbouring a rifampicin resistance-conferring variant were included (282/312). We further excluded samples originating from Tanzania and Botswana, due to missing or unknown clinical data, and 3 other samples with incomplete clinical data, resulting in a final dataset of 203 rifampicin-resistant strains used for the univariable and multivariable analysis. The 203 samples used are depicted in the supplementary table 1.

Phylogenetic reconstruction: A maximum likelihood phylogeny was inferred with RAxML v.8.2.8 using an alignment containing only polymorphic sites and excluding the variable positions in drug resistance-related genes. The phylogeny was inferred using the general time-reversible model of sequence evolution and *Mycobacterium canettii* (SRR011186) was used as outgroup to root the phylogeny. The R package ggtree v1.12.7 (9) was used to plot the phylogeny and annotate it.

Compensatory mutations: Compensatory mutations in *rpoA/C* described by Comas et al (1) and Merker et al (10) were used to parse the *M. tuberclusosis* genomes.

Figure S1. Distribution of different groups of isoniazid resistance-conferring mutations by HIV status.

Group 1 is *katG* mutations other than S315T, group 2 is *katG* S315T mutations and group 3 is

inhA promoter mutations. X-squared = 1.2269, df = 2, p-value = 0.5415.

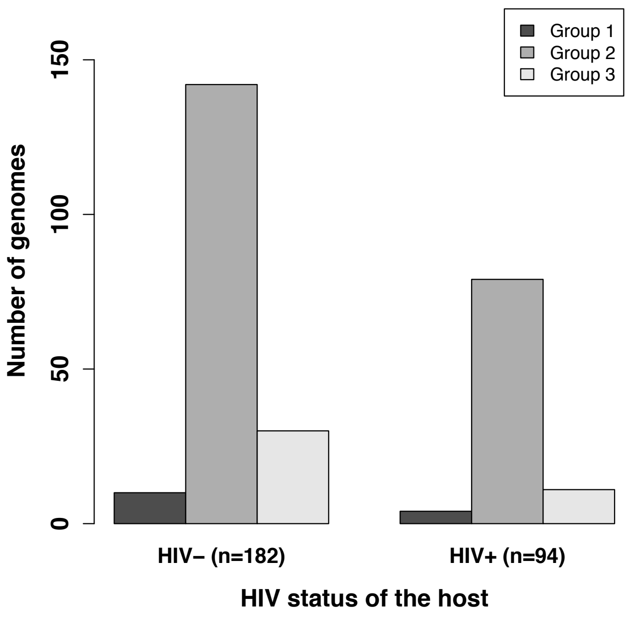

References

1. Zürcher K, Ballif M, Fenner L, Borrell S, Keller PM, Gnokoro J, Marcy O, Yotebieng M, Diero L, Carter EJ, Rockwood N, Wilkinson RJ, Cox H, Ezati N, Abimiku AG, Collantes J, Avihingsanon A, Kawkitinarong K, Reinhard M, Hömke R, Huebner R, Gagneux S, Böttger EC, Egger M. 2019. Drug susceptibility testing and mortality in patients treated for tuberculosis in high-burden countries: a multicentre cohort study. Lancet Infect Dis 19:298–307.
2. Van Embden JDA, Cave MD, Crawford JT, Dale JW, Eisenach KD, Gicquel B, Hermans P, Martin C, McAdam R, Shinnick TM, Small PM. 1993. Strain identification of Mycobacterium tuberculosis by DNA fingerprinting: Recommendations for a standardized methodology. J Clin Microbiol 31:406–409.
3. Bolger AM, Lohse M, Usadel B. 2014. Trimmomatic: A flexible trimmer for Illumina sequence data. Bioinformatics 30:2114–2120.
4. Li H, Durbin R. 2009. Fast and accurate short read alignment with Burrows-Wheeler transform. Bioinformatics 25:1754–1760.
5. Mckenna A, Hanna M, Banks E, Sivachenko A, Cibulskis K, Kernytsky A, Garimella K, Altshuler D, Gabriel S, Daly M, DePristo MA. 2010. The Genome Analysis Toolkit: a MapReduce framework for analyzing next-generation DNA sequencing data. Genome Res 20:1297–1303.
6. Li H. 2011. A statistical framework for SNP calling, mutation discovery, association mapping and population genetical parameter estimation from sequencing data. Bioinformatics 27:2987–2993.
7. Koboldt DC, Zhang Q, Larson DE, Shen D, McLellan MD, Lin L, Miller CA, Mardis ER, Ding L, Wilson RK. 2012. VarScan 2: Somatic mutation and copy number alteration discovery in cancer by exome sequencing. Genome Res 22:568–576.

8. Cingolani P, Platts A, Wang LL, Coon M, Nguyen T, Wang L, Land SJ, Lu X, Ruden DM. 2012. A program for annotating and predicting the effects of single nucleotide polymorphisms, SnpEff: SNPs in the genome of Drosophila melanogaster strain w1118; iso-2; iso-3. Fly (Austin) 6:80–92.
9. Yu G, Smith DK, Zhu H, Guan Y, Lam TTY. 2017. Ggtree: an R Package for Visualization and Annotation of Phylogenetic Trees With Their Covariates and Other Associated Data. Methods Ecol Evol 8:28–36.
10. Merker M, Barbier M, Cox H, Rasigade JP, Feuerriegel S, Kohl TA, Diel R, Borrell S, Gagneux S, Nikolayevskyy V, Andres S, Nübel U, Supply P, Wirth T, Niemann S. 2018. Compensatory evolution drives multidrug-resistant tuberculosis in central Asia. Elife 7:1–31.
